# Is Gout Associated with Pyoderma Gangrenosum? A Population-Based Case-Control Study

**DOI:** 10.3390/jcm9061626

**Published:** 2020-05-28

**Authors:** Khalaf Kridin, Ralf J. Ludwig, Dana Tzur Bitan, Mouhammad Kridin, Giovanni Damiani, Arnon D. Cohen

**Affiliations:** 1 Lübeck Institute of Experimental Dermatology, University of Lübeck, 23562 Lübeck, Germany; Ralf.Ludwig@uksh.de; 2 Department of Behavioral Sciences, Ariel University, 40700 Ariel, Israel; dana.tzur@gmail.com; 3 Sackler School of Medicine, Tel-Aviv University, 6997801 Tel-Aviv, Israel; 7amada.k@gmail.com; 4 Clinical Dermatology, IRCCS Istituto Ortopedico Galeazzi, 20161 Milan, Italy; dr.giovanni.damiani@gmail.com; 5 Department of Biomedical, Surgical and Dental Sciences, University of Milan, 20122 Milan, Italy; 6 Clalit Health Services, 6264705 Tel-Aviv, Israel; ardcohen@gmail.com

**Keywords:** pyoderma gangrenosum, gout, case-control study

## Abstract

The coexistence of pyoderma gangrenosum (PG) and gout has been reported in individual patients; however, the association between these conditions has not been investigated. We aimed to assess the association between PG and gout and to examine whether the presence of gout predisposes to the development of PG. A population-based case-control study was conducted comparing PG patients (*n* = 302) with age-, sex-, and ethnicity-matched control subjects (*n* = 1497) with respect to the presence of preceding gout. Logistic regression models were utilized for univariate and multivariate analyses. The prevalence of preceding gout was greater in patients with PG than in control subjects (3.7% vs. 0.7%, respectively; *p* < 0.001). Gout was associated with a more than fivefold increase in the risk of PG (OR, 5.15; 95% CI, 2.21–11.98). After adjusting for confounding factors, gout emerged as a significant independent predictor of PG (adjusted OR, 4.08; 95% CI, 1.69–9.80). Gout preceded the diagnosis of PG by a median latency of 4.6 years. Patients with gout-associated PG were older, predominantly male, and had a higher prevalence of metabolic syndrome than other patients with PG. In conclusion, gout increases the risk of developing PG by more than fivefold. Physicians managing patients with gout and PG should be aware of this emerging association.

## 1. Introduction

Pyoderma gangrenosum (PG) is a rare, neutrophilic, inflammatory, and ulcerative dermatosis present typically with erythematous nodules or sterile pustules that rapidly evolve into very painful ulcerations. The characteristic ulcer of PG forms violaceous undermined borders on the lower extremities, although it may affect other anatomical locations as well as peristomal regions [1]. PG is associated with underlying systemic conditions in 57% of patients [2]. A better characterization of underlying diseases is highly important because the type and severity of these comorbidities are of prognostic significance for PG [3,4]. Gout is a metabolic disease characterized by the deposition of needle-like crystals of monosodium urate in the synovial fluid and other tissues. The clinical manifestations of gout include gouty arthritis, tophi resulting from the accumulation of crystals in connective tissue, uric acid nephrolithiasis, and renal impairment [5].

The coexistence of PG with gout has been reported anecdotally in a few case reports and case series [6,7,8]; however, the association between these conditions is yet to be investigated by controlled epidemiological studies, leaving the literature inconclusive in this regard.

The aim of the current study is to investigate the association between gout and PG and to assess whether the presence of gout predisposes individuals to developing PG. Moreover, we aim to delineate the epidemiological features of patients with gout-associated PG relative to other patients with PG.

## 2. Methods

### 2.1. Study Design and Database

We conducted a population-based case-control study aiming to evaluate the risk of developing subsequent PG following the diagnosis of gout. To fit this study design, only patients in whom the outcome (PG) followed the exposure (gout) were included in the analysis.

The current study was grounded on the computerized database of Clalit Health Services (CHS). Ensuring 4,927,000 enrollees as of October 2018, CHS is the principal health care organization in Israel, providing healthcare services for 57% of the general Israeli population (based on the 2019 census of the Central Bureau of Statistics). CHS offers an inclusive array of both public and private healthcare services and possesses a multitude of primary, secondary, and tertiary healthcare facilities throughout the whole country. The computerized database of CHS was built using uninterrupted real-time data input from clinical, pharmaceutical, and administrative operating systems. Providing data that enabled the performance of multiple epidemiological studies, this database was proven very effective and reliable.

One of the main resources of the CHS database is the chronic diseases registry. It retrieves data from various sources, including hospital and primary care reports, claims of medications and health services utilization, and laboratory and imaging data. These data are then individually cross-checked and validated by the primary care physician of each patient. Given the multiple strata of verifications, this registry was shown to be of high validity and reproducibility [9].

The current study was approved by the institutional review board (IRB) of Ben-Gurion University in accordance with the declaration of Helsinki (approval code: 0212-17-COM).

### 2.2. Study Population

All individuals with a diagnostic code consistent with the diagnosis of PG between the years 2000 and 2018 were identified. Subsequently, cases were checked and were considered eligible if one of the following criteria was met: (i) a documented diagnosis of PG registered at least twice by a community board-certified dermatologist, and/or (ii) documentation of the diagnosis of PG in hospital discharge letters from dermatological wards.

The diagnosis of gout was based on its documentation in the chronic registry of CHS. Therefore, it was based on its registration by board-certified rheumatologists, the purchase of gout-related medications, suggestive laboratory and imaging data, and eventually its authentication by the managing general healthcare provider.

The control group encompassed up to five control individuals per patient, matched randomly by age, sex, and ethnicity. The date of diagnosis of each patient represented the index date, both for the patient and for the corresponding matched controls. Before their enrollment, controls were confirmed as being alive and contributing longitudinal data for the CHS dataset.

### 2.3. Study Variables and Sensitivity Analyses

Outcome measures were controlled for underlying comorbidities as assessed by the Charlson comorbidity index, a validated epidemiological method of quantifying comorbidities. This index has been evidenced to be reliable in predicting lethal outcomes [10]. Outcomes were also adjusted for metabolic syndrome, owing to its established triggering effect in gout [11]. Metabolic syndrome was defined as the presence of at least three of the following conditions: type 2 diabetes mellitus, dyslipidemia, hypertension, or obesity [12], as was previously demonstrated [13]. Smoking status was classified as either current smoker or never/past smoker. The adjustment for smoking was performed owing to the conflicting evidence regarding its impact on predisposing inflammatory bowel disease-associated PG [14,15,16,17].

Two sensitivity analyses were held to increase the validity of our findings. The first was carried out to confirm that the observed association was not overestimated due to ascertainment bias. This analysis was based on repeating the calculations after excluding patients diagnosed with gout up to two years before PG and recruitment in cases and controls, respectively. Given that rheumatoid arthritis (RA) is among the most frequent comorbidities in patients with PG, and it may bear a clinical overlap with gout [2], a second sensitivity analysis excluded cases with coexistent gout and RA. This avoided the misclassification of RA as gout in light that gout may clinically mimic RA. Only one case of concomitant gout and RA was found.

### 2.4. Statistical Analysis

Baseline characteristics were described by means and standard deviations (SDs) for continuous variables, whilst categorical values were signified by percentages. A comparison of sociodemographic and clinical factors between cases and controls was performed using the Chi-square test and *t*-test.

Logistic regression was used to calculate odds ratios (ORs) and 95% CIs to compare cases and controls regarding the presence of gout. In the case-control analysis, the association was calculated based on individuals who developed PG following the diagnosis of gout in accordance with the presence of a temporal relationship between exposure and outcome. In the cross-sectional sub-analysis, all patients with both diagnoses were included. Two-tailed *p*-values less than 0.05 were considered as statistically significant. All statistical analyses were performed using SPSS software, version 25 (SPSS, IBM Corp, Armonk, NY, USA).

## 3. Results

### 3.1. Characteristics of the Study Population

The study population encompassed 302 patients with PG and 1497 matched control individuals. The mean (SD) age at the diagnosis of patients and recruitment of control subjects was 54.0 (20.8) years. One-hundred-seventy-five (57.9%) patients with PG were females, and 255 (84.4%) were of Jewish ethnicity. The two groups were comparable with regard to sex distribution, ethnic composition, mean body mass index (BMI), and the prevalence of smoking, as shown in Table 1. The mean (SD) Charlson comorbidity score was greater in cases than in controls (2.3 (2.7) vs. 1.3 (1.8), respectively; *p* < 0.001). Severe comorbidities were more prevalent among cases relative to controls (37.4% vs. 19.2%, respectively; *p* < 0.001). The characteristics of the study population are delineated in Table 1.

### 3.2. Case-Control Study Design

The prevalence of preceding gout was greater among patients with PG than among control subjects (3.7% vs. 0.7%, respectively; *p* < 0.001). A more than fivefold increase in the odds of PG was observed with preceding gout (OR, 5.15; 95% CI, 2.21–11.98). Stratification by age, sex, and ethnicity led the association between gout and subsequent PG to be more prominent among younger (<54 years; OR, 10.13; 95% CI, 0.91–112.50), male (OR, 5.33; 95% CI, 2.07–13.71), and Jewish (OR, 5.75; 95% CI, 2.31–14.30) patients, respectively. The association was only of marginal statistical significance among female patients (OR, 5.02; 95% CI, 0.70–35.90). In a stratified analysis based on smoking status, the association was only significant among non-smokers (OR, 7.20; 95% CI, 2.47–21.01), as shown in Table 2.

A sensitivity analysis, excluding patients diagnosed with gout up to two years before PG, was carried out. The association of gout and later PG retained its statistical significance (OR, 4.63; 95% CI, 1.87–11.49). Another sensitivity analysis excluded patients with a coexistent diagnosis of RA and gout and revealed that the association between gout and subsequent PG was not altered substantially (OR, 4.68; 95% CI, 1.97–11.12). Of note, only one case had coexistent gout and RA.

Additionally, we performed a multivariate analysis, adjusting for putative confounding factors like BMI, smoking, and metabolic syndrome. The association between gout and later PG was proven independently significant in this analysis (adjusted OR, 4.08; 95% CI, 1.69–9.80; *p* = 0.002). Metabolic syndrome was also significantly associated with gout, as shown in Table 3.

### 3.3. Cross-Sectional Study Design

The lifetime prevalence of gout was greater among patients with PG than among control subjects (4.6% vs. 1.0%, respectively; OR, 4.80; 95% CI, 2.29–10.06; *p* < 0.001). When a stratified analysis was performed, the association between PG and gout was still significant among males and individuals older than 54 years of age, whilst a marginal statistical significance emerged in younger and female patients. On the other hand, the association also became significant among smokers, as shown in Table 4.

### 3.4. Temporal Relationship between the Two Investigated Conditions

Among patients with coexistent PG and gout (*n =* 14), the diagnosis of PG followed that of gout in the majority of patients (78.6%; *n* = 11). In cases where PG followed the onset of gout, the mean (SD) latency between the conditions was 5.5 (4.3) years, whereas the median (range) was 4.6 (0.1–14.8) years. Of these patients, 72.7% (*n* = 8) developed PG at least three years after the diagnosis of gout.

In three patients (21.4%), the diagnosis of PG preceded that of gout by a mean (SD) latency of 4.4 (2.0) years. The median (range) latency between the diagnosis of PG and the development of gout was 3.1 (2.8–7.3) years.

### 3.5. The Clinical Characteristics of Patients with Gout-Associated PG as Compared to Other Patients with PG

We then compared the epidemiological features of patients with gout-associated PG (*n* = 14) as compared to the remaining patients with PG (*n* = 288). Patients with a dual diagnosis of PG and gout were significantly older at the onset of PG (69.1 (11.2) vs. 53.3 (20.9) years, respectively; *p* = 0.005), had a prominent male preponderance (78.6% vs. 40.3%, respectively; *p* = 0.005), greater prevalence of metabolic syndrome (64.3% vs. 26.0%, respectively; *p* = 0.002), and a higher mean (SD) Charlson comorbidity score (5.5 (3.1) vs. 2.2 (2.6), respectively; *p* = 0.002). The ethnic background, BMI, and frequency of smoking were comparable between the two subgroups, as shown in Table 5.

## 4. Discussion

The current case-control study revealed that a preceding diagnosis of gout is associated with a fivefold increase in the risk of subsequent PG. This association was stronger among males and patients of Jewish ancestry and was robust to two sensitivity analyses and to multivariate analysis controlling for confounders. The median latency between the diagnosis of gout and the development of PG was 4.6 years. Patients with gout-associated PG were older, had more associated comorbidities, and greater prevalence of metabolic syndrome.

It has long been known that PG is associated with underlying systemic comorbidities in a considerable number of patients. This concept was substantiated by a recent meta-analysis that summarized the literature with respect to the frequency and distribution of underlying systemic diseases among patients with PG. Synthesizing data across 21 studies encompassing 2611 patients with PG, this meta-analysis was able to disclose that 57% of patients with PG had an underlying systemic disease at the onset of PG. Inflammatory bowel disease, inflammatory arthritis (mainly RA), hematological malignancies (mainly monoclonal gammopathies), and solid malignancies were the most frequently encountered comorbidities [2].

Gout is typified by several cutaneous manifestations. Attacks of acute gouty arthritis may leave a violaceous hue on the skin, often followed by desquamation. More characteristically, cutaneous deposits of monosodium urate, coined as “tophi”, may appear during the course of chronic tophaceous gout as firm dermal or subcutaneous papules and nodules [5]. Gouty panniculitis is another rare cutaneous manifestation of gout arising from deposits of monosodium urate crystals in the lobular hypodermis. The latter morphologically manifests as tender erythematous, subcutaneous nodules, or plaques with an irregular surface [18].

Gout was found to associate with PG in scattered case reports and case series. Cabalag et al. [6] found that 3 out of 29 patients (10.3%) with PG had preceding gout in an Australian case series. Ye and Ye [8] reported that 1 out of their 23 patients (4.3%) with PG had underlying gout. However, the onset of PG in this 82-year-old male patient was attributed to the presence of concomitant monoclonal gammopathy. Another case report depicted a patient with coexistent PG and gout responding favorably to ustekinumab [7]. Despite this partial knowledge, no observational studies were performed to investigate whether a true epidemiological association exists between these diseases. Elucidating the spectrum of comorbid diseases in PG is of great clinical implication, since early identification of comorbid diseases may attenuate the disease course and result in better response to treatment [19].

The pathomechanism underlying the observed association is unknown; however, several mechanistic explanations could be hypothesized. It has been shown that deposits of urate crystals can activate the NLRP3 (cryopyrin) inflammasome and stimulate the production of the active proinflammatory cytokine interleukin (IL)-1β by monocytes and macrophages [20]. The overproduction of IL-1β triggers the release of several proinflammatory cytokines and chemokines, resulting in the recruitment and activation of neutrophils [21]. The latter may lead to a neutrophil-mediated inflammation, which is the pathophysiological hallmark of PG [22].

Although not directly related to the etiopathogenesis of the disease, patients with gout were found to synthesize several autoantibodies against different antigens [23,24]. Similarly, patients with PG experienced a disruption in humoral immunity, leading to the overproduction of circulating autoantibodies. Previous diagnostic criteria had even specified the presence of various circulating autoantibodies as a minor diagnostic criterion of PG [22]. Taken together, the increase in circulating autoantibodies may be a potential explanation for the observed association. Further research is required to establish these hypotheses.

Another putative explanation of the observed association is that gout may arise from adverse events derived from PG-related medications, mainly corticosteroids, leading to weight gain and metabolic syndrome, which embodies a risk factor of gout [11]. The fact that PG followed gout in the vast majority of cases argues against this notion. Additionally, the case-control study design only took cases in which PG followed gout into consideration, and still demonstrated an increased association, thus refuting the aforementioned interpretation.

The current study provides a novel epidemiological knowledge and sheds light on a topic that has not been previously investigated. Including patients from primary, secondary, and tertiary healthcare settings renders the study less susceptible to selection bias. The case-control design enables us to identify the temporal sequence in which the investigated diseases appeared, thus allowing us to estimate the risk of PG based on the assumption that the OR approximates relative risk in case-control studies of rare diseases [25]. Nonetheless, the absence of data regarding the morphological features and severity of the investigated diseases is the main drawback of the current study. These features, as well as their effects on the reported associations, should be examined in future studies. The low number of patients with a dual diagnosis of PG and gout represent a major drawback of the current study.

To conclude, the current case-control population-based study reveals epidemiological evidence regarding a robust association between gout and PG. Patients with preceding gout are at a more than fivefold increase in the risk of developing PG. This risk was particularly prominent among males, Jewish patients, and non-smokers. The association held significance following multivariate analysis adjusting for confounders, including metabolic syndrome. A median latency of 4.6 years separated the diagnosis of gout and the emergence of PG. Relative to other patients with PG, patients with gout-associated PG were older, had higher male preponderance, greater comorbidities, and an increased prevalence of metabolic syndrome. Physicians managing patients with gout may be aware of this emerging association. Dermatologists encountering patients with rapidly evolving ulcers may inquire about a history of gout. Further research is required to establish this observation in other study populations.

## Figures and Tables

**Table 1:** Descriptive characteristics of the study population.

Characteristic	Patients with Pyoderma Gangrenosum (*n* = 302)	Controls (*n =* 1497)	*p* Value
Age, years			
Mean ± SD	54.0 ± 20.8	54.0 ± 20.8	1.000
Median (range)	55.8 (0.2–95.1)	55.9 (0.2–95.6)	
Male sex, *n* (%)	157 (57.9%)	629 (58.0%)	0.974
Ethnicity, *n* (%)			
Jews	255 (84.4%)	1264 (84.4%)	1.000
Arabs	47 (15.6%)	233 (15.6%)	
BMI, mg/kg^2^			
Mean ± SD	28.0 ± 6.3	27.8 ± 6.2	0.614
Smoking, *n* (%)	115 (38.1%)	521 (34.8%)	0.274
Charlson comorbidity score			
Mean score ± SD	2.3 ± 2.7	1.3 ± 1.8	**<0.001**
None (0)	111 (36.8%)	777 (51.9%)	**<0.001**
Moderate (1–2)	78 (25.8%)	432 (28.9%)	0.276
Severe (≥3)	113 (37.4%)	288 (19.2%)	**<0.001**

Abbreviations: *n*, Number; SD, standard deviation; BMI, body mass index. Bold: significant value.

**Table 2:** The risk of pyoderma gangrenosum (PG) in patients with a preceding diagnosis of gout stratified by age, sex, and ethnicity (a case-control design).

Subgroup	Number	Gout in Patients with PG (*n =* 299) *n* (%) *	Gout in Controls (*n =* 1494) *n* (%) *	OR (95% CI)	*p* Value
All	1792	11 (3.7%)	11 (0.7%)	**5.15 (2.21–11.98)**	**<0.001**
Age, years					
<54	840	2 (1.4%)	1 (0.1%)	**10.13 (0.91–112.50)**	**0.020**
≥54	952	9 (5.7%)	10 (1.3%)	**4.70 (1.88–11.76)**	**<0.001**
Gender					
Male	752	9 (7.2%)	9 (1.4%)	**5.33 (2.07–13.71)**	**<0.001**
Female	1040	2 (1.1%)	2 (0.2%)	5.02 (0.70–35.90)	0.074
Ethnicity					
Jews	1513	10 (4.0%)	9 (0.7%)	**5.75 (2.31–14.30)**	**<0.001**
Arabs	279	1 (2.1%)	2 (0.9%)	2.50 (0.22–28.15)	0.443
Smoking status					
Non-smokers	1160	8 (4.3%)	6 (0.6%)	**7.20 (2.47–21.01)**	**<0.001**
Smokers	632	3 (2.7%)	5 (1.0%)	2.84 (0.67–12.04)	0.140

* The prevalence of gout in cases when gout preceded PG (in cases) or preceded recruitment (in controls). Abbreviations: PG, pyoderma gangrenosum; OR, odds ratio; *n*, Number; CI, confidence interval. Bold: significant value.

**Table 3:** The association between gout and the later development of PG: Multivariate analysis *.

Variable	Adjusted OR	95% CI	*p* Value
Pyoderma gangrenosum	4.08	1.69–9.80	**0.002**
Smoking	1.02	0.41–2.49	0.975
Body mass index	1.00	0.99–1.02	0.773
Metabolic syndrome	5.68	2.26–14.27	**<0.001**

* Adjusting for all the covariates listed in Table 3. Abbreviations: CI, confidence interval. Bold: significant values.

**Table 4:** The association between pyoderma gangrenosum and gout, stratified by age, sex, and ethnicity (a cross-sectional design).

Subgroup	Number	Gout in Patients with PG (*n =* 302) *n* (%) *	Gout in Controls (*n =* 1497) *n* (%) *	OR (95% CI)	*p* Value
All	1799	14 (4.6%)	15 (1.0%)	**4.80 (2.29–10.06)**	**<0.001**
Age, years					
<54	841	2 (1.4%)	2 (0.3%)	5.07(0.71–36.27)	0.073
≥54	958	12 (7.4%)	13 (1.6%)	**4.82 (2.16–10.77)**	**<0.001**
Gender					
Male	756	11 (8.7%)	11 (1.7%)	**5.33 (2.26–12.58)**	**<0.001**
Female	1043	3 (1.7%)	4 (0.5%)	3.77 (0.84–16.98)	0.064
Ethnicity					
Jews	1519	13 (5.1%)	12 (0.9%)	**5.61 (2.53–12.43)**	**<0.001**
Arabs	280	1 (2.1%)	3 (1.3%)	1.67 (0.17–16.38)	0.658
Smoking status					
Non-smokers	1163	8 (4.3%)	9 (0.9%)	**4.80 (1.83–12.61)**	**<0.001**
Smokers	636	6 (5.2%)	6 (1.2%)	**4.73 (1.50–14.93)**	**0.004**

* Lifetime prevalence of gout regardless of whether it preceded or followed PG. Abbreviations: PG, pyoderma gangrenosum; OR, odds ratio; *n*, Number; CI, confidence interval. Bold: significant value.

**Table 5:** Comparison between patients with gout-associated pyoderma gangrenosum relative to the remaining patients with pyoderma gangrenosum.

	Gout-Associated PG (*n* = 14)	PG without Gout (*n* = 288)	*p* Value
Age at the onset of PG, years; mean (SD)	69.1 (11.2)	53.3 (20.9)	**0.005**
Male sex, *n* (%)	11 (78.6%)	116 (40.3%)	**0.005**
Jewish ethnicity, *n* (%)	13 (92.9%)	228 (79.2%)	0.213
Body mass index, kg/m^2^, mean (SD)	30.5 (5.0)	27.8 (6.3)	0.070
Smokers, *n* (%)	6 (42.9%)	109 (37.8%)	0.702
Metabolic syndrome, *n* (%)	9 (64.3%)	75 (26.0%)	**0.002**
Charlson Comorbidity Score; mean ± SD	5.5 ± 3.1	2.2 ± 2.6	**0.002**

Abbreviations: PG, pyoderma gangrenosum; *n*, number; SD, standard deviation. Bold: significant value.
